# Nanomaterial‐Enhanced Electrochemical Sensors for Clinical Monitoring of Acyclovir: Integration Into Molecular Diagnostics

**DOI:** 10.1002/jcla.70104

**Published:** 2025-09-30

**Authors:** Mohammad Darvishi, Mohammad Mahdi Heidari, Reza Kheradmand, Nava Moghadasian Niaki, Mahsa Tabean, Ahmad Mobed

**Affiliations:** ^1^ Infectious Disease, School of Aerospace and Subaquatic Medicine, Infectious Diseases & Tropical Medicine Research Center(IDTMC), AJA University of Medical Sciences Tehran Iran; ^2^ Department of Pediatrics, School of Medicine Iran University of Medical Sciences Tehran Iran; ^3^ Social Determinants of Health Research Center, Health Management and Safety Promotion Tabriz Iran; ^4^ Women's Reproductive Health Research Center Tabriz University of Medical Sciences Tabriz Iran

**Keywords:** acyclovir, biosensor, electrochemical sensors, nanomaterial‐based sensor, pharmaceutical drug

## Abstract

**Objective:**

This study investigates the innovative application of nanomaterial‐based micro‐devices aimed at enhancing the diagnosis and management of acyclovir (ACV) for herpes virus infections, specifically those caused by HSV‐1, HSV‐2, CMV, and VZV.

**Background:**

Herpes viruses are associated with various clinical diseases, highlighting the urgent need for effective antiviral therapies. Acyclovir remains a primary treatment option; however, its potential for kidney toxicity and allergic reactions necessitates careful dosage monitoring, particularly in immunocompromised patients.

**Methods:**

Recent advancements in drug monitoring systems have been developed to optimize dosing regimens and reduce the risk of misuse. This study focuses on the integration of biological and electrochemical nanosensors utilizing nanomaterials, which have shown significant improvements in detection capabilities and diagnostic sensitivity for ACV.

**Results:**

We delineate the novel features and applications of these micro‐devices, emphasizing their unique configurations and unprecedented limits of detection.

**Conclusion:**

This research illustrates how these advanced technologies can enhance existing methodologies in herpes virus management, ultimately leading to improved treatment outcomes.

## Graphical abstract

Schematic illustration of biosensor technology for detection of ACV.

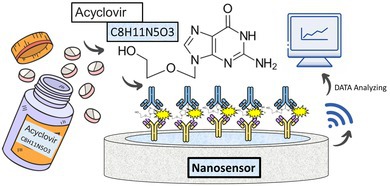

**Abbreviations**
CPE: carbon paste electrode
DHP: dihexadecyl hydrogen phosphate
DPV: differential pulse voltammetry
GCE: glassy carbon electrode
GE: gold electrode
LSV: linear sweep voltammetry
MBZ/TMHPP Cu(II)/GE: a gold electrode (GE) modified with a self‐assembled monolayer of 2‐mercaptobenzothiazol (MBZ) and tetrakis(3‐methoxy‐4‐hydroxyphenyl)porphyrinato‐copper(II)(TMHPPtetrakis(3‐methoxy‐4‐hydroxyphenyl)porphyrinato‐copper(II) (TMHPP Cu(II)))
MWNTs: multi‐wall carbon nanotubes
MWNTs‐DHP/GCE: a multi‐wall carbon nanotubes (MWNTs)‐Dihexadecyl(MWNTs)–dihexadecyl hydrogen phosphate (DHP) film‐coated GCE
NC: nano clay
PEBT: poly‐eriochrome black T
PS:Beta‐CDPS: Beta‐CD IC/Y_2_O_3_/GCE: polystyrene: Beta‐cyclodextrin inclusion complex‐supported yttrium oxide (Y2O3)‐modified GCE
PVP/CPE: polyvinylpyrrolidone (PVP) modified CPE
rGO: reduced graphene oxide
SPGE: screen‐ printed graphite electrode
SWV: square wave voltammetry
UTGE: ultra trace graphite electrode
ZnO/GCE: ZnO nanoparticles modified GCE

## 1 Introduction

Infectious diseases remain a significant global health challenge, with various pathogens contributing to morbidity and mortality [1, 2, 3]. Among these, the herpes virus family, which includes Herpes simplex virus type 1 (HSV‐1), Herpes simplex virus type 2 (HSV‐2), cytomegalovirus (CMV), and varicella‐zoster virus (VZV), is particularly impactful [4, 5]. These viruses are associated with a wide spectrum of clinical manifestations, which can vary significantly in severity. In some cases, individuals may experience mild symptoms such as localized itching, redness, or discomfort, which can often be mistaken for other conditions. Conversely, these viruses can also lead to more severe illnesses, including extensive skin lesions, systemic infections, and complications affecting vital organs, particularly in immunocompromised individuals [6]. Effective management of herpes virus infections relies on antiviral therapies, with acyclovir (ACV) being a cornerstone treatment because of its efficacy in targeting viral replication [6]. The significance of herpes viruses extends beyond immediate health concerns; certain members of this family, such as Epstein–Barr virus (EBV) and CMV, are implicated in cancer development, highlighting the need for effective management strategies in oncology [7]. Additionally, HSV‐2 poses serious risks in gynecology and obstetrics, particularly concerning neonatal herpes, which can have life‐threatening implications for newborns. Therefore, accurate dosing of antiviral medications is crucial for optimizing therapeutic outcomes, especially in vulnerable populations [8]. Recent advancements in drug monitoring systems have underscored the importance of accurately assessing medication levels to enhance treatment efficacy and prevent complications. In this context, electrochemical sensors have emerged as innovative tools for real‐time drug monitoring [9, 10, 11]. The integration of nanomaterials into these sensors offers significant advantages, including improved detection sensitivity and enhanced electron transfer, which are vital for the effective monitoring of antiviral drugs like ACV [9, 12]. This study aims to explore the features and applications of contemporary nanomaterial‐based sensors in the context of monitoring and diagnosing medications. By focusing on their potential to enhance the diagnosis and management of antiviral therapies, this research seeks to contribute to the ongoing efforts to improve therapeutic strategies for viral infections and optimize patient outcomes.

## 2 Herpes Viruses

**FIGURE 1:**
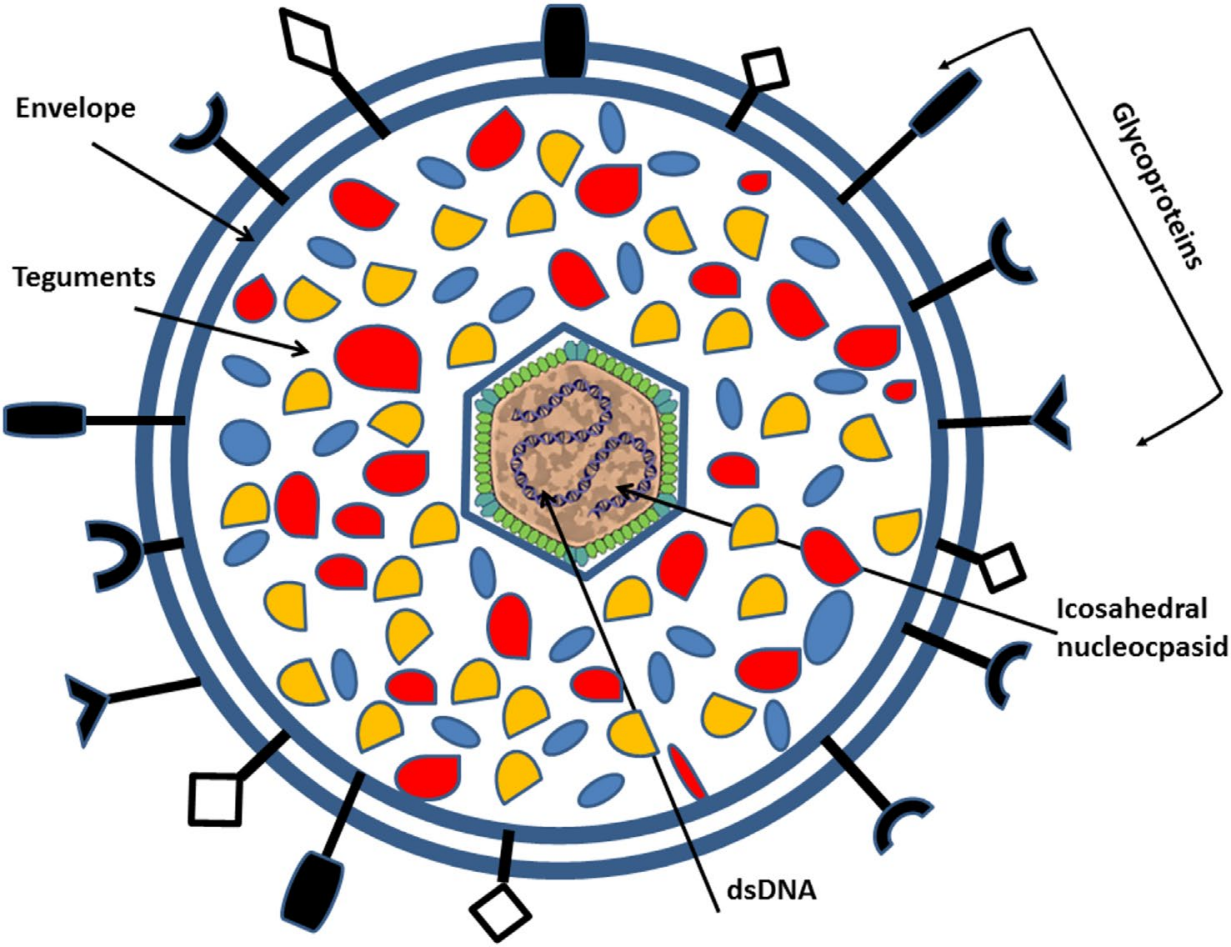
Schematic structure of herpes viruses.

Herpes viruses, including herpes simplex viruses (HSV‐1 and HSV‐2), varicella‐zoster virus, and cytomegalovirus, are significant human pathogens responsible for a wide range of infections [9]. The Herpesviridae family consists of large, double‐stranded DNA viruses that can cause various diseases. HSV‐1 and HSV‐2 are particularly prevalent, with approximately 67% and 13% of the global population seropositive, respectively [9]. Infections can be asymptomatic or lead to serious conditions such as cold sores, genital herpes, and herpes simplex encephalitis. The interaction between herpes viruses and the host immune system is crucial in determining the severity of the infection, with genetic factors influencing susceptibility, especially in neonates, who are at higher risk for severe outcomes because of their immature immune systems [13]. Transmission of herpes viruses occurs through various routes, including saliva, skin lesions, respiratory droplets, sexual contact, and from mother to child during childbirth or breastfeeding. Although infection with one type of HSV typically induces immunity against re‐infection with the same type, it does not provide protection against other types [14]. Herpes viruses possess distinct characteristics, including an icosahedral structure, a double‐stranded DNA genome, and the ability to persist in the host indefinitely. They encode numerous proteins and enzymes, including DNA polymerase, which are critical targets for antiviral therapies. Understanding the biology and transmission of these viruses is essential for developing effective prevention and treatment strategies [15]. HHVs establish a life‐long infection in the host and exist as either a latent phase or a lytic phase (i.e., viral replication stage). In the initial infection and the lytic phase, herpes viruses cause various diseases in infected individuals. After the initial infection, these viruses maintain a latent state in healthy individuals. The infection state is switched from the latent phase to the lytic phase by viral reactivation. The lytic phase is induced under conditions of UV exposure, immunodeficiency, drug administration, or hormonal changes [16]. During the latent phase of infection, the cellular reservoir is sensory ganglia neurons for alpha‐herpes viruses [17], bone marrow progenitors (the myeloid lineage) for HCMV, HHV6A, and HHV6B, CD4 T cells for HHV7 [18, 19], and B lymphocytes for gamma‐herpes viruses [20]. The cytopathologic effect, DNA sequence similarity, replicative cycle (short or long), and site of latent infection are involved in the family classification of human herpes viruses (HHVs) into three subfamilies. On the basis of their biological characteristics, herpes viruses are divided into three subgroups called alpha (α), beta (β), and gamma (γ) herpes viral, as shown in Figure 1 [17].

Chemotherapeutic agents for viral diseases are classified into three main categories: virucides, antivirals, and immune response modifiers. Virucides are substances that inhibit virus transmission and include organic solvents, detergents, nanoparticles, and ultraviolet light [21]. Antivirals are essential for treating viral infections and can also serve as preventive measures, referred to as suppressive or pre‐emptive therapy. Most antivirals have a narrow spectrum, specifically targeting certain viruses [21]. Currently, more than 40 antiviral agents are FDA‐approved, with many developed recently for the treatment of HIV. These medications function by inhibiting viral replication; however, their excessive use may compromise the body's ability to fight off other viral infections. ACV is one of the key medications used against the herpes virus.

## 3 Acyclovir

**FIGURE 2:**
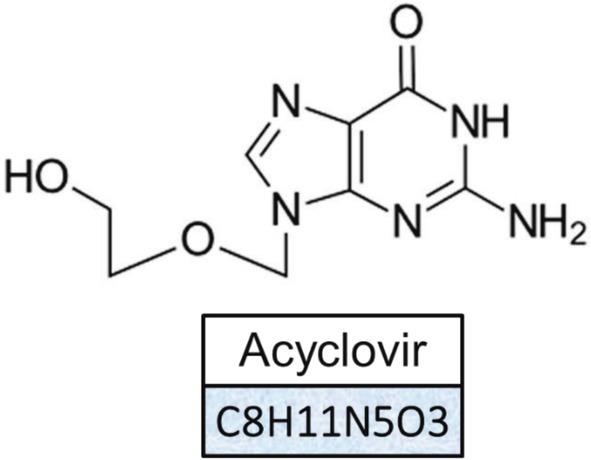
Acyclovir [2‐amino‐9‐((2‐hydroxyethoxy) methyl)‐1H‐purin‐6(9H)‐one].

ACV, along with ganciclovir (GCV) and penciclovir (PCV), is widely used to treat infections caused by the herpes simplex virus [22]. These compounds work by inhibiting viral replication, which helps to halt the progression of the infection and alleviate symptoms, ultimately shortening recovery time and reducing the risk of transmission to others [22]. ACV is a guanine nucleoside analogue and is one of the most commonly prescribed antiviral medications globally. It is considered safe and can be administered intravenously, orally, or topically with minimal side effects [23]. Despite its safety profile, excessive use of ACV can lead to neurotoxicity, headaches, renal issues, and gastrointestinal disturbances. ACV has low water solubility and a short half‐life in the body, with only 15%–20% of the drug being metabolized; the majority is excreted unchanged in urine [24]. High doses are typically required for effective treatment, such as 200 mg five times daily for genital herpes in adults and children over 12, although higher doses are recommended for neonatal herpes simplex virus infections. ACV is effective against various viral illnesses, including hepatitis B and herpes viruses like HSV, varicella‐zoster virus (VZV), and Epstein–Barr virus (EBV), with minimal impact on normal cells. It is FDA‐approved for treating genital herpes and HSV encephalitis, and although it is also used for conditions like mucocutaneous HSV, herpes zoster, and chickenpox, these uses are not FDA‐approved [25]. ACV remains the first‐line treatment for HSV encephalitis, although there has yet to be a systematic review assessing its efficacy for this specific condition. The ACV chemical structure is presented in Figure 2.

To enhance the effectiveness of current antiviral therapies utilizing ACV, there is a need for new or improved techniques and formulations [26]. Strategies such as less frequent dosing, reduced ACV dosages, and sustained‐release mechanisms can help optimize treatment while minimizing the risk of side effects associated with high drug concentrations [26]. These advancements are likely to improve patient adherence to antiviral regimens involving ACV. Recent research has focused on nanoparticulate systems as drug carriers, garnering significant global interest [27]. This innovative approach is considered a promising advancement in antiviral therapy, as nanoparticles can deliver medications effectively while minimizing toxicity. Given the potential toxicological and adverse effects of ACV, it is crucial to monitor and analyze its levels in the body [27]. Numerous analytical methods have been developed to accurately identify and quantify ACV not only in commercial pharmaceutical products but also in human urine and serum. This monitoring plays a vital role in ensuring the safe use of the drug in patients [28]. In conclusion, the lytic life cycle of human herpesviruses is a complex and highly coordinated process that enables these viruses to efficiently infect host cells and replicate. From the initial binding to host cell receptors to the final egress of new virions, each step is crucial for the successful propagation of the virus. Understanding this life cycle not only provides insights into the fundamental biology of herpesviruses but also highlights potential targets for therapeutic intervention. By disrupting specific stages of the viral life cycle, such as entry, gene expression, or DNA replication, researchers can develop antiviral strategies that may effectively mitigate the impact of herpesvirus infections. Continued exploration of these mechanisms will be essential for advancing our knowledge and improving treatment options for diseases caused by human herpesviruses. Monitoring and determining the appropriate dosage of ACV present several challenges that can impact its efficacy and safety in clinical use. One significant challenge is the variability in individual patient responses, which can be influenced by factors such as age, weight, renal function, and the presence of coexisting medical conditions. This variability necessitates careful consideration of dosing regimens to avoid underdosing, which may lead to treatment failure, or overdosing, which can increase the risk of adverse effects. Additionally, the accurate measurement of ACV levels in biological fluids can be complicated by the drug's pharmacokinetics, including its rapid distribution and elimination. The presence of other medications and potential drug interactions further complicates monitoring efforts, as they can alter acyclovir's metabolism and excretion. Consequently, healthcare providers must employ a combination of clinical judgment, therapeutic drug monitoring, and patient‐specific factors to optimize ACV therapy and ensure effective antiviral treatment.

## 4 Significance of Nanoscale and Sensitive Detection of ACV in Antiviral Therapy

The nanoscale and sensitive determination of ACV is important for several reasons. First, ACV is an antiviral medication used primarily to treat infections caused by certain types of viruses, such as herpes simplex and varicella‐zoster [29]. Accurate measurement of its concentration in biological fluids is crucial for therapeutic drug monitoring to ensure efficacy and minimize toxicity. Understanding the pharmacokinetics of ACV, including its absorption, distribution, metabolism, and excretion, requires sensitive detection methods [30]. Nanoscale techniques can provide detailed insights into how the drug behaves in the body. Additionally, sensitive determination methods can help in the early detection of viral resistance to ACV, which is critical for effective treatment planning and management of viral infections [31]. In the development of new formulations, such as nanoparticles or liposomes for improved delivery of ACV, nanoscale analysis is essential to evaluate the drug's stability, release profile, and bioavailability. Furthermore, nanoscale techniques can facilitate research into new derivatives or analogs of ACV, potentially leading to more effective antiviral agents with improved properties. In pharmaceutical manufacturing, sensitive determination methods are necessary for quality control to ensure that ACV products meet regulatory standards for potency and purity. Overall, the ability to detect ACV at the nanoscale with high sensitivity enhances our understanding of its pharmacological properties and improves patient care through better monitoring and treatment strategies.

## 5 ACV Detection Methods

Because of the excessive usage and large dosage of ACV, many other side effects and certain toxicity hazards will occur in animals. There are many analytical methods that can effectively quantify and detect ACV in commercial pharmaceutical formulations, human urine, and serum. Spectrophotometry is a technique used for both qualitative and quantitative analysis of substances, and it remains the preferred method for routine analytical tasks. Many colorless substances that do not absorb visible light can be converted into colored compounds through chromogenic reactions, allowing for their detection via spectrophotometry. This process enhances the sensitivity and selectivity of the analysis. There are several types of chromogenic reactions, including complexation, redox, and condensation reactions. Consequently, the application of spectrophotometry for the assay of ACV is well‐supported and valid [32]. Research has shown that ACV undergoes an oxidative coupling reaction with 3‐methylbenzothiazolin‐2‐one hydrazone (MBTH) in the presence of hydrochloric acid (HCl) and the Fe (III) oxidant, resulting in a deep‐green colored product. This method is straightforward, innovative, and reliable for detecting ACV in pharmaceutical formulations, with a limit of detection (LOD) of 1.06 μg/mL and an analysis wavelength of 616 nm. Additionally, ACV can react with various other substances to form new molecules, which can also be analyzed using spectrophotometry [33]. A study found that the primary amino group of ACV can undergo a condensation and coupling reaction with ninhydrin–ascorbic acid in a citric acid buffer at pH 5, resulting in the formation of a purple chromophore known as Ruhemann's purple. At the maximum absorption wavelength of 540 nm, there was a strong linear relationship between the concentration of ACV and the intensity of color development, leading to the establishment of a photometric method for ACV determination. After optimizing the reaction conditions and conducting interference tests, this method demonstrated high sensitivity (LOD: 0.3 μg/mL) and good selectivity, making it suitable for quality control of ACV in hospitals and laboratories [34]. One study created an indirect approach for determining ACV using spectrophotometry. Essentially, an excess of N‐bromo‐succinimide (NBS) was introduced into an acidic solution to oxidize ACV. The remaining NBS was then utilized to decolorize a fixed quantity of methyl orange. The measurement of ACV was conducted indirectly by assessing the absorbance of methyl orange at 508 nm, with a LOD of 0.2 μg/mL. This technique leverages the interactions between ACV, NBS, and methyl orange, presenting a viable spectrophotometric method for ACV analysis [35]. High performance liquid chromatography (HPLC), a chromatographic analysis method using liquid as the mobile phase, is one of the chromatography‐based techniques used to analyze ACV, generally involving different detectors. Typically, ultraviolet–visible (UV–VIS) [36, 37, 38], fluorescence [39, 40], photodiode array (PDA), and diode‐array detector (DAD) [41] are coupled with it. Octadecyl (C18) and monomer octyl (C8) stationary phases are commonly used for efficient packing for reversed phase separations [32].

**FIGURE 3:**
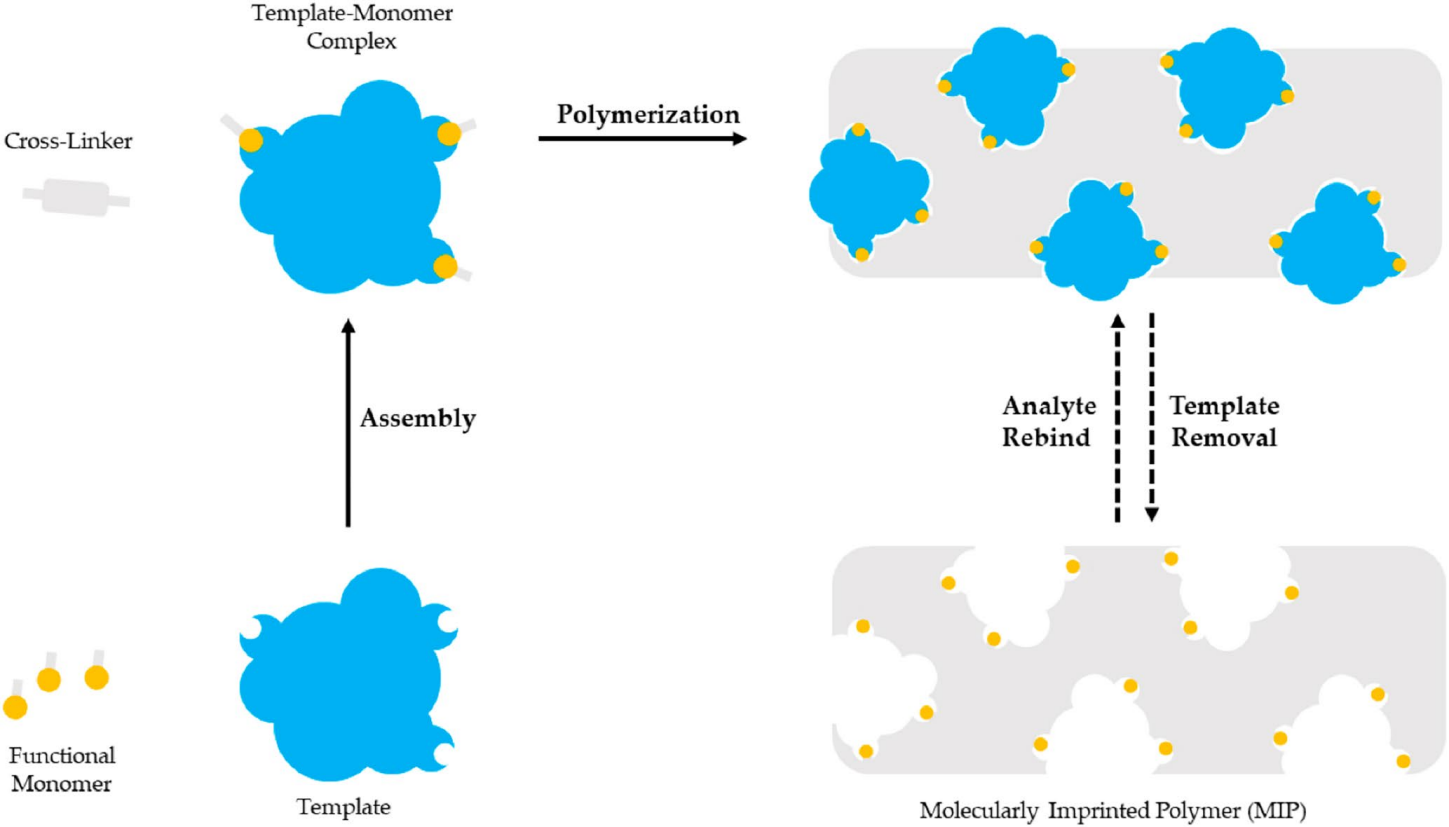
Schematic illustration of MIP methodology [46].

One of the primary difficulties in quantifying ACV using HPLC is that the detected peaks can be easily obscured, and the retention times tend to be relatively lengthy. To address this issue, researchers investigated optimal detection conditions for measuring ACV across different settings. They utilized UV detection at a wavelength of 255 nm on a reversed‐phase C8 column, employing a mobile phase consisting of 0.1% (v/v) triethylamine in water at pH 2.5 to facilitate the detection of ACV in plasma samples. The sample preparation involved precipitating proteins with 20% (v/v) perchloric acid, which effectively separated the ACV peak from those of interfering endogenous compounds. This approach was successfully modified for analyzing the pharmacokinetic profiles of ACV tablets [42]. Liquid chromatography/tandem mass spectrometry (LC–MS/MS) is a chromatographic method that merges the high separation capabilities of liquid chromatography with the exceptional sensitivity and specificity of mass spectrometry. Likewise, its operational principles necessitate specific types of mobile phases and other conditions to function effectively [43]. The key distinction is that mass spectrometers are utilized to address spectral interferences that can occur with PDA/UV–Vis detectors. Given the technique's sensitivity and specificity/selectivity, LC–MS/MS is often preferred for precise measurements. Currently, quadrupole tandem mass spectrometry is the most commonly used form of tandem mass spectrometry [32]. As technology advances, LC–MS/MS systems are continually being refined and enhanced. High‐resolution mass spectrometry (HRMS) not only provides precise measurements of ion masses but also accurately identifies their elemental and isotopic compositions [44]. Time of flight mass spectrometry (TOF), serving as the second mass analyzer, has emerged as a significant area of development because of its exceptional resolution, broad mass range, rapid scanning capabilities, and high sensitivity. Additionally, ultra‐performance liquid chromatography (UPLC) offers benefits such as increased analytical throughput, enhanced sensitivity, and greater peak capacity by leveraging the principles of HPLC along with innovative technologies, including rapid detection methods [45]. Molecular imprinting is a prospective technology to synthesize molecularly imprinted polymers (MIPs) (Figure 3) that have specific cavities matched with the target molecule. At present, with the advantages of low cost, simple preparation, being more environmentally friendly, specificity, affinity, high selectivity, and having high stability for the target analyte, MIPs are widely used in chemical sensors, solid‐phase extraction, artificial antibodies, and other fields [47].

**FIGURE 4:**
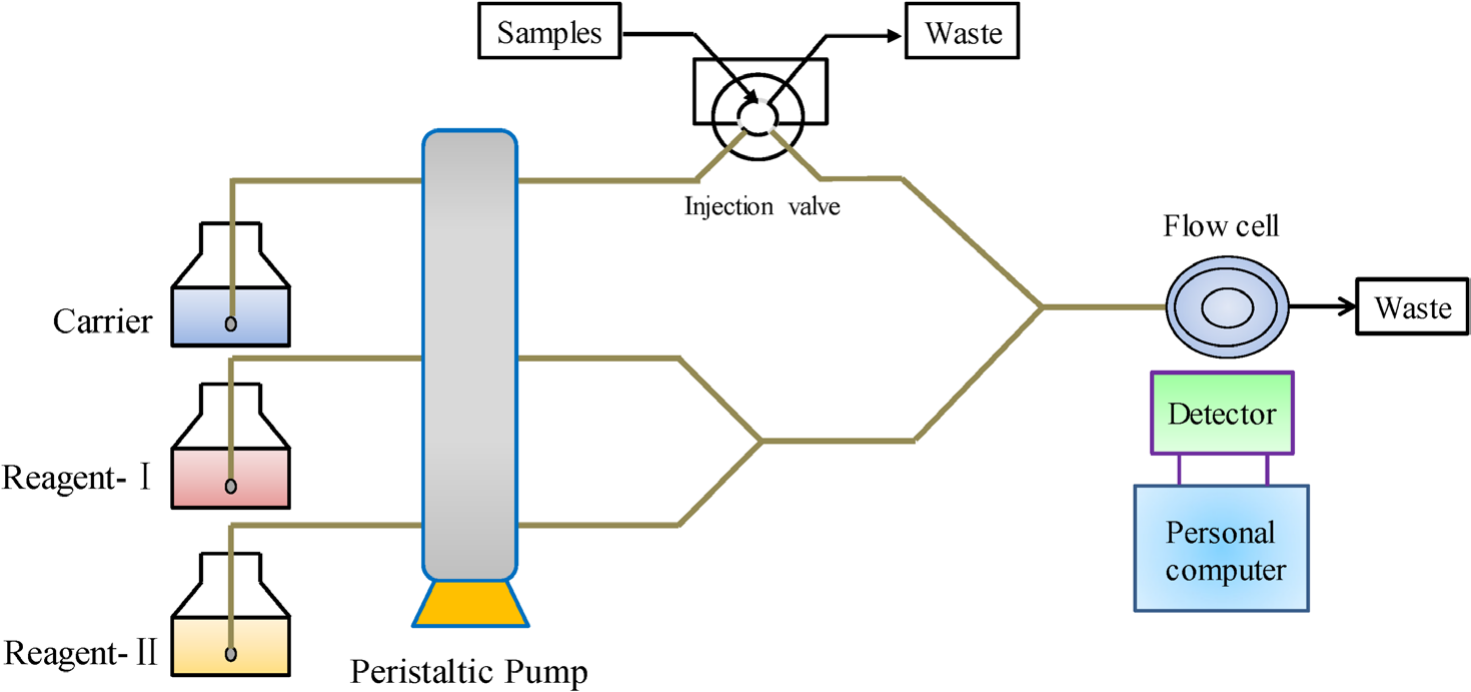
Schematic diagram of the FI–CL analysis system [32].

An unbalanced structure of molecularly imprinted polymers (MIPs) often arises from local temperature fluctuations during exothermic polymerization, leading to uneven polymer morphology. To address this, researchers developed a novel method to create homogeneous MIPs by mimicking the multiple hydrogen bonds found in nucleotide bases. This involved grafting onto silica supports using ACV as a template molecule, resulting in a balanced MIP structure. The process began with activating silica microspheres to create vinylated silica, which were then polymerized with ACV and allyl‐cytosine using the initiator 2,2′‐azobisisobutyronitrile (AIBN). After purifying the solid polymers and removing ACV, molecularly imprinted microspheres (MIMs) were produced. These MIMs were effectively used for solid‐phase extraction in conjunction with high‐performance liquid chromatography (HPLC) to capture and detect ACV in serum samples, achieving a LOD of 1.8 ng/mL and a mean recovery rate of 95.6% [48]. A common approach to creating a molecular imprint involves using methacrylic acid (MAA) as the functional monomer, ethylene glycol dimethacrylate (EGDMA) as the crosslinking agent, and azobisisobutyronitrile (AIBN) as the initiator. Research has indicated that miniaturized molecularly imprinted solid‐phase extraction (mini‐MISPE) combined with high‐performance liquid chromatography (HPLC) can be utilized for the analysis of ACV in urine samples [49]. Flow injection–chemiluminescence (FI‐CL) is a highly effective analytical method that integrates chemiluminescence (CL) analysis with a flow injection approach [50]. Because of its benefits, including high sensitivity, broad linear range, straightforward instrumentation, and ease of use, it is commonly employed in various fields such as environmental monitoring, pharmaceutical analysis, and food testing [51]. A schematic diagram of the FI–CL analysis system is presented in Figure 4.

In an alkaline medium, ACV significantly inhibits Ni(IV) complex‐luminol chemiluminescence (CL) systems, showing a linear correlation between the level of inhibition and ACV concentration within a specific range. A study developed a Ni(IV) complex‐luminol CL system for ACV detection using flow injection chemiluminescence (FI‐CL). Although Ni(IV) complexes react with luminol to form a luminescent intermediate, they do not react with ACV. Instead, the luminescent intermediates transfer some free radicals to ACV, decreasing the free radicals of luminol and thus reducing the system's luminescence intensity. The results indicated a strong linear relationship between CL intensity and ACV concentration in the range of 50–1200 μg/L, with a LOD of 0.03 mg/L and satisfactory stability [32]. At low concentrations, ACV can enhance the chemiluminescence (CL) of the luminol–H_2_O_2_ system in an alkaline medium, with CL intensity directly proportional to ACV concentration within the range of 0.09–3 μmol/L. This method demonstrated a low LOD of 77.1 nmol/L and a low relative standard deviation (RSD) of 0.43% over 11 parallel tests at 1.00 μmol/L, proving effective for ACV determination. Additionally, researchers discovered that ACV reacts with potassium permanganate to produce CL in the presence of formaldehyde in an acidic solution. They developed a new flow injection chemiluminescence (FI‐CL) method for ACV detection, which exhibited high selectivity, a wide linear range of 0.2–80 mg/L, and a low LOD of 0.06 mg/L [52]. Table 1 presents a summary of the analytical methods used for detecting ACV.

**TABLE 1:** Summary of Analytical Methods for Detecting ACV.

Method	Operating wavelength (nm)	Linear range (μg/L)	LOD	Recovery (%)	Samples	Ref
Spectrophotometry (MBTH reaction)	616	1.06 μg/mL	1.06 μg/mL	Not specified	Pharmaceutical formulations	[10]
Spectrophotometry (Ninhydrin)	540	Not specified	0.3 μg/mL	High sensitivity	Quality control in hospitals/labs	[12]
Spectrophotometry (NBS Method)	508	Not specified	0.2 μg/mL	Not specified	Indirect determination of ACV	[13]
High‐performance liquid chromatography (HPLC)	255	Not specified	Not specified	Not specified	Plasma samples	[21]
Liquid chromatography/tandem mass spectrometry (LC–MS/MS)	Not specified	Not specified	Not specified	Not specified	Not specified	[22]
Molecularly imprinted polymers (MIPs)	Not specified	Not specified	1.8 ng/mL	95.6%	Serum samples	[27]
Flow injection–chemiluminescence (FI‐CL)	Not specified	50–1200 μg/L	0.03 mg/L	Not specified	Not specified	[10]
Flow injection–chemiluminescence (H_2_O_2_ System)	0.09–3 μmol/L	0.09–3 μmol/L	77.1 nmol/L	Not specified	Not specified	[52]

## 6 Challenges and Alternative Solutions for Traditional ACV Detection

Traditional methods for detecting ACV often face several challenges that can impact their effectiveness in clinical settings [53]. One significant limitation is sensitivity; many conventional detection techniques, such as spectrophotometry and chromatography, may not achieve the sensitivity required to detect low concentrations of ACV, particularly in complex biological matrices [53]. This limitation can lead to inaccurate assessments of drug levels, especially in immunocompromised patients who require precise dosing. Another challenge is specificity. Traditional methods may struggle with specificity, as they can be influenced by the presence of other substances in the sample. This can result in false positives or negatives, complicating the interpretation of results and potentially leading to inappropriate treatment decisions [54]. Additionally, biological samples often contain a variety of compounds that can interfere with the detection of ACV. For instance, metabolites, proteins, and other drugs may affect the accuracy of traditional assays, necessitating extensive sample preparation and purification steps. Furthermore, many traditional detection methods require lengthy sample preparation and analysis times, which can delay clinical decision‐making and patient management [55]. To address these limitations, several alternative solutions have emerged, leveraging advancements in technology. The integration of nanomaterials in sensor design has shown great promise in enhancing the sensitivity and specificity of ACV detection. These sensors can provide rapid and accurate measurements, even at low concentrations, because of their high surface area and unique electrochemical properties. Additionally, biosensors that utilize biological recognition elements, such as enzymes or antibodies, can offer high specificity for ACV [42]. These devices can be designed to provide real‐time monitoring of drug levels, allowing for timely adjustments in therapy. Moreover, advanced analytical techniques such as mass spectrometry and HPLC coupled with advanced detection methods can significantly improve the sensitivity and specificity of ACV detection. These methods can also reduce the impact of interfering substances, providing more reliable results. Emerging technologies, including wearable sensors and point‐of‐care testing devices, enable real‐time monitoring of ACV levels in patients [56]. This capability is particularly beneficial for managing therapy in immunocompromised individuals, where precise dosing is critical. By exploring these alternative solutions, we can enhance the detection and monitoring of ACV, ultimately improving patient outcomes and the management of herpes virus infections. Recent advancements in nanosensors for antiviral surveillance have significantly enhanced the ability to detect and monitor viral infections, including those treated with antiviral agents like ACV. These nanosensors leverage the unique properties of nanomaterials, such as their high surface area and sensitivity, to provide rapid and accurate detection of viral biomarkers in various biological samples. Innovations in sensor design, including the integration of nanocomposites and functionalized nanoparticles, have improved the specificity and sensitivity of these devices, allowing for the identification of low viral loads that traditional methods may miss. Furthermore, the development of portable and user‐friendly nanosensor platforms facilitates point‐of‐care testing, enabling timely diagnosis and monitoring of viral infections in diverse settings, from clinical environments to remote locations. As research continues to evolve, the potential for nanosensors to provide real‐time surveillance of antiviral efficacy and viral resistance is becoming increasingly promising, paving the way for more effective management of viral diseases and improved patient outcomes.

## 7 Biosensor

**FIGURE 5:**
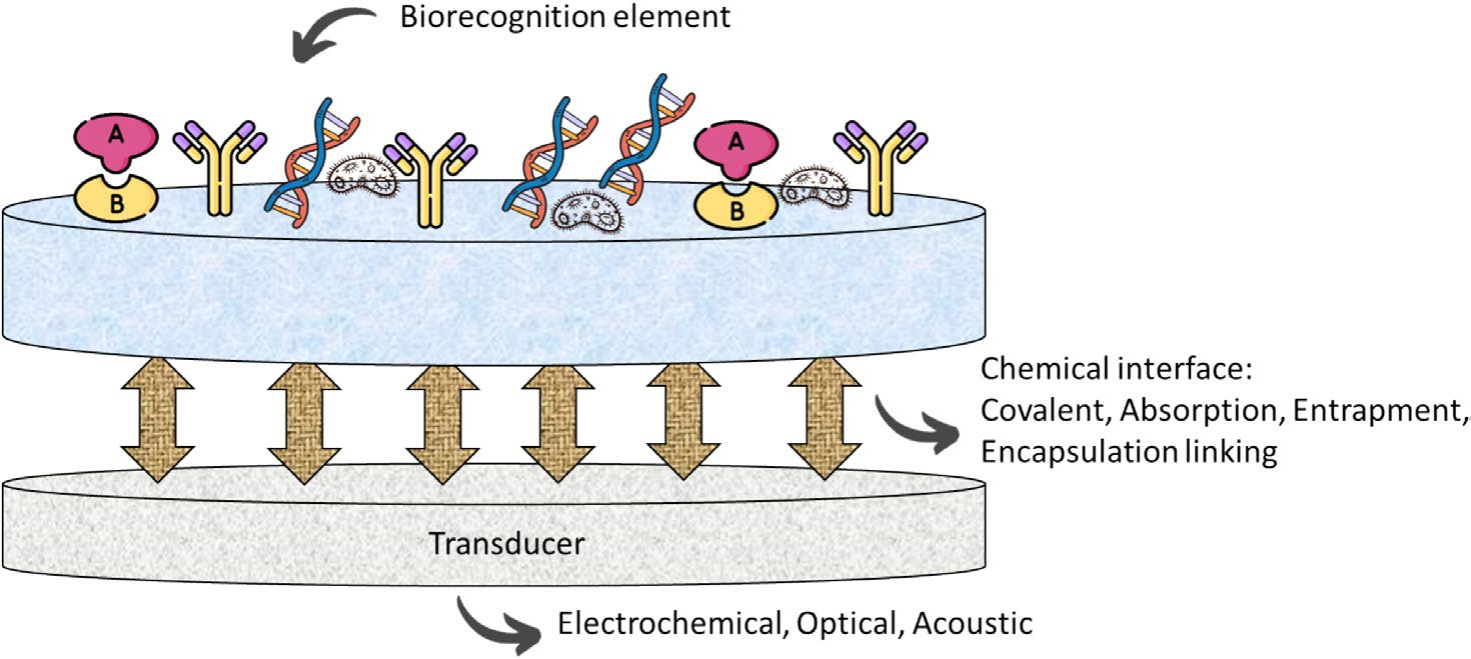
Biosensor structure.

A biosensor (Figure 5) is a sophisticated device designed to convert chemical information from biomolecule concentrations into meaningful analytical signals [57, 58]. These devices find extensive applications across various sectors, including medical diagnostics, food safety, process control, and environmental monitoring [57, 58]. The fundamental structure of a biosensor comprises four key components: sensing elements (or receptors) that specifically bind to the target analyte; an interface that creates an optimal working environment for the biosensor elements; a transducer that converts the physical or chemical signals generated from the interaction between the sensing elements and the analyte into electrical signals; and electronic components for signal amplification, processing, and data analysis [59]. Biosensors are built on a sensor matrix that is functionalized to attach biorecognition elements (BREs) for sensitive analyte detection. The choice of material, fabrication method, and design of the sensor matrix significantly affect the sensor's performance and must be tailored to the specific analyte and transduction mechanism. Common sensor matrices include paper, graphite, carbon paste, glassy carbon electrodes, screen‐printed electrodes, and indium tin oxide [60]. The BREs are immobilized on the transducer surface to selectively interact with analyte molecules, allowing for quantification of the biorecognition event for further applications. BREs can be categorized into biocatalytic elements like enzymes and bio‐affinity elements such as antibodies, nucleic acids, and aptamers [61]. Alternatively, nanomaterial‐modified surfaces can be employed for sensing because of their catalytic properties, although they may lack the selectivity of traditional BREs. The selection of BREs involves balancing sensitivity, selectivity, reproducibility, reusability, and ease of fabrication. Transducers play a crucial role in converting biorecognition events into measurable signals, and biosensors can be classified on the basis of the type of transducer into optical, mechanical, or electrochemical categories [62]. Additionally, biosensors can be categorized by receptor type (biocatalytic, immunological, or nucleic acid), transduction physics (electrochemical, optical, piezoelectric, or thermal), and application fields (medical, environmental, or wearable). Commercial biosensors are further divided into laboratory‐based and portable types [62, 63]. Optical biosensors detect changes in phase, polarization, or frequency in the optical field because of analyte interaction and can be further classified into absorption, fluorescence, and luminescence‐based sensors. Absorption‐based biosensors measure changes in light amplitude when analytes are present, with colorimetric biosensors being particularly useful in low‐resource settings because of their simplicity and reliance on visual detection [64]. Mechanical biosensors quantify changes in mechanical parameters such as force and motion following biomolecular interactions. They offer several advantages, including high mass resolution for detecting minute analyte quantities, rapid sensing times for studying biological events, and the ability to detect analytes in both liquid and gas phases without the need for labeling, which is often required in optical sensing.

Electrochemical biosensors operate by measuring changes in current, potential, conductance, or field effect resulting from the interaction between target molecules and biorecognition elements on their sensing surfaces [65, 66]. They are favored for their robustness, selectivity, sensitivity, and overall analytical performance compared to other types of biosensors. These biosensors typically utilize a three‐electrode system, which includes a working electrode (such as a glassy carbon electrode, GCE, that serves as the transducing element), a counter electrode (like a platinum electrode that completes the circuit), and a reference electrode (such as an Ag/AgCl electrode that maintains a stable potential) [65, 66]. Selecting the appropriate working electrode is crucial, as it undergoes nanomaterial modification and surface engineering. The operation of electrochemical biosensors is based on the kinetics of ion or electron transfer from the reaction site to the electrode surface. Both label‐free and labeled electrochemical biosensors measure variations in electron transfer. They can be categorized into different types on the basis of the transduction method: potentiometric, amperometric/voltammetric, and impedimetric/conductometric. In amperometric and voltammetric biosensors, a variable or static potential is applied to the working electrode relative to the reference electrode, and the resulting current is measured [67]. This potential triggers redox reactions of electrolytes at the working electrode, generating electrons that produce a current response proportional to the analyte concentration. Various electrochemical techniques, such as cyclic voltammetry and differential pulse voltammetry, utilize different combinations of potential, current, charge, and time for analysis [68]. Potentiometric biosensors measure the potential of an electrochemical cell at the working electrode when no current flows, providing insights into the redox activities within the cell. These sensors often incorporate biorecognition elements like enzymes to detect catalytically generated ions. Conductometric biosensors assess changes in the electrical conductivity of a solution as its composition varies during a chemical reaction [69]. Impedimetric biosensors measure the impedance between the working and counter electrodes relative to the reference electrode. This method is particularly useful for label‐free detection, as it does not require detection tags. The binding of analytes to a nanomaterial‐modified surface reduces electron transfer, increasing the system's impedance, which can be calibrated using known analyte concentrations [70]. Biological and electrochemical nanosensors offer significant advantages in detecting ACV because of their unique properties. These nanosensors enhance sensitivity, enabling the detection of ACV at very low concentrations, which is critical for early diagnosis in clinical settings. Their use of biological recognition elements, such as antibodies and enzymes, ensures high selectivity, reducing interference from other compounds. Additionally, nanosensors facilitate rapid electron transfer and mass transport, resulting in quicker response times compared to traditional methods. This capability is essential in dynamic biological environments where ACV concentrations can vary, allowing for real‐time monitoring that supports timely therapeutic decisions. The small size of these sensors makes them ideal for portable diagnostic devices, improving accessibility to ACV detection in various settings, including remote areas. Their integration into handheld devices promotes point‐of‐care testing, enhancing patient management. Moreover, the versatility of biological and electrochemical nanosensors allows them to utilize different detection methods, optimizing strategies on the basis of specific needs. Their compatibility with advanced technologies, such as microfluidics and lab‐on‐a‐chip systems, enables the development of integrated platforms that can conduct multiple analyses simultaneously, streamlining the detection process and providing comprehensive data for informed treatment decisions. In summary, the use of biological and electrochemical nanosensors represents a significant advancement in ACV detection, effectively addressing the challenges previously discussed. Their sensitivity, rapid response, portability, versatility, and integration capabilities highlight their potential as a promising solution in nanotechnology for effective monitoring and management of ACV.

## 8 Nanomaterials in Biosensors

Nanomaterials play a crucial role in the fabrication of biosensors, enhancing their performance by facilitating rapid ion movement between electrodes and target molecules, improving electro‐catalytic activities, enabling fast analyte diffusion, concentrating analyte molecules near the electrode surface, and providing anti‐fouling properties [71]. Various nanomaterials with different sizes, shapes, and properties are utilized to achieve specific objectives in biosensors, often by functionalizing them to conjugate with biorecognition elements for bio‐affinity or biocatalytic applications [71]. Metallic nanoparticles, particularly gold nanoparticles (AuNPs), are widely used in biosensing because of their unique size‐dependent optical and electronic properties, chemical inertness, and biocompatibility. AuNPs can be combined with other nanomaterials or conjugated with antibodies or aptamers to create bio‐affinity biosensors [72]. Carbon‐based nanomaterials, such as carbon nanotubes (CNTs), graphene oxide (GO), and fullerenes, are also prevalent in biosensing because of their excellent conductivity and catalytic activity [73]. Graphene and its derivatives are especially valued for their superior electrical properties and ease of functionalization. Luminescent semiconducting nanocrystals, known as quantum dots (QDs), are employed in both optical and electrochemical biosensors for their photo‐electrochemical behavior. Additionally, conducting polymers like polypyrrole and poly(3,4‐ethylenedioxythiophene) (PEDOT) are low‐cost, flexible, and biocompatible materials used in electrochemical biosensors because of their functionalization potential and electrical conductivity [73, 74]. The engineering of the nano‐surface of sensing probes is vital for maintaining the integrity of sensing layers, as it involves creating a nano‐bio‐interface where biorecognition elements are immobilized to interact with analytes. The stability and consistency of this attachment are crucial for generating reliable signals across various physiological conditions [75]. Functionalization strategies for nanomaterials can be classified into covalent methods (like click chemistry) and non‐covalent interactions (such as electrostatic forces). Although physisorption is a simpler technique for immobilization, it is sensitive to environmental changes, making stronger covalent interactions preferable for biosensor development [76]. Recent advancements in electrochemical analysis technology have expanded its application in pharmaceutical analysis, including drug quality control and toxic substance detection. Electrochemical methods offer advantages over traditional techniques, such as simplicity, high sensitivity, and rapid in situ detection [77]. Various functional materials, including graphene and metal nanoparticles, can enhance the performance of electrochemical sensors by amplifying signals and improving interactions with target molecules. For instance, researchers have developed a polypyrrole‐coated multi‐walled CNT (MWCNT) sensor for detecting ACV, which demonstrated high redox activity and sensitivity [77]. Other modified materials, such as γ‐Fe_2_O_3_ nanoparticles combined with bentonite clay, have also been used to enhance the performance of carbon paste electrodes for ACV detection, achieving a low limit of quantitation. Various electrode types, including pencil graphite and glassy carbon electrodes, have been employed for ACV assays, showcasing the versatility and effectiveness of nanomaterials in biosensing applications. Nanomaterials provide significant advantages in sensor development, especially in electrochemical applications, because of their small size, high sensitivity, and selectivity. Their unique properties, such as large surface area and excellent conductivity, enhance interactions with analytes, improving drug detection. CNTs are particularly valued for their superior conductivity and ability to facilitate electron transfer and organic molecule adsorption, leading to enhanced detection performance. Single‐walled CNTs (SWCNTs) further improve electron transport because of their small size and structure [78]. Graphene, known for its exceptional electrical and structural properties, is beneficial for electrochemical detection. Although oxygen groups in graphene oxide can enhance analyte detection, they may also reduce conductivity. These groups can be selectively reduced to create defects that accelerate catalysis. Doping graphene with heteroatoms enhances its properties, making it suitable for cost‐effective electrode materials that improve sensor sensitivity and selectivity. Noble metal nanomaterials are commonly used for detecting pharmaceutical analytes because of their reactivity and diverse forms. Bi‐ or trimetallic nanoparticles can enhance detection through synergistic effects, and various three‐dimensional nanomaterials can serve as effective detection platforms. Metal‐carbon matrix composites are being explored for their high conductivity and cost‐effectiveness, with nanoparticles adhering to carbon materials to increase sensitivity [79]. TiO_2_‐supported nanostructures are also popular in electrochemical detection because of their stability and low toxicity, with metal nanoparticles enhancing their properties [80]. Lastly, conductive polymers have emerged as promising materials for sensor applications, improving selectivity and sensitivity. Atomically imprinted polymer (MIP) composites are particularly effective in complex organic environments, addressing challenges related to selectivity and fouling. Nanomaterials have shown great promise in the detection of ACV because of their unique properties, which enhance sensitivity and specificity in analytical applications. Various types of nanomaterials, such as gold, silver, and QD nanoparticles, can be functionalized to selectively bind to ACV, allowing for colorimetric or fluorescence‐based detection. Additionally, CNTs and graphene oxide can be utilized in electrochemical sensors to improve conductivity and enhance the detection signal of ACV. Detection mechanisms involving nanomaterials include colorimetric detection, where nanoparticles change color in the presence of ACV, providing a visual indication of its concentration. For instance, gold nanoparticles can aggregate in the presence of ACV, leading to a color change that can be quantified. Fluorescence detection is another method, where QDs create fluorescent sensors that emit light at specific wavelengths when ACV is present, allowing for highly sensitive detection. Electrochemical sensors also benefit from nanomaterials, as their incorporation can enhance the electrochemical response. CNTs, for example, increase the surface area and conductivity, resulting in improved detection limits for ACV. The advantages of using nanomaterials for ACV detection include high sensitivity, rapid response times, and selectivity. The large surface area‐to‐volume ratio of nanomaterials enables the detection of low concentrations of ACV, whereas their ability to provide quick results is crucial for clinical applications. Furthermore, the functionalization of nanomaterials can be tailored to selectively bind to ACV, reducing interference from other substances. Surface functionalization strategies, such as the attachment of specific ligands or antibodies, can significantly enhance the specificity of the detection process, ensuring that only ACV is targeted while minimizing cross‐reactivity with other compounds. Applications of nanomaterial‐based sensors extend to clinical diagnostics, where they can be used for the rapid detection of ACV levels in patient samples, aiding in therapeutic drug monitoring. These sensors can also be applied to environmental monitoring, detecting ACV in wastewater or environmental samples to assess the impact of pharmaceutical contaminants. However, there are challenges to consider, such as ensuring the long‐term stability and reproducibility of nanomaterial‐based sensors for practical applications. Additionally, for clinical use, these detection methods must undergo rigorous testing and regulatory approval. Integrating nanomaterial sensors with existing diagnostic platforms can further enhance their utility and accessibility. In summary, nanomaterials offer innovative approaches for the detection of ACV, with potential applications in both clinical and environmental settings. Their unique properties can lead to the development of highly sensitive, rapid, and selective detection methods, particularly when combined with effective surface functionalization strategies to enhance specificity.

## 9 ACV Biosensors

A clay‐like, two‐dimensional transition metal carbide, Ti_3_C_2_, was produced by etching Ti_3_AlC_2_ with LiF and HCl. Carboxylated (MWCNTs‐COOH) were integrated with Ti_3_C_2_ using chitosan (CS) as a solvent and subjected to ultrasonic treatment for the detection of ACV. This nanocomposite was subsequently applied to the surface of a GCE to create an electrochemical sensor for ACV detection [81]. An MIP was employed to fabricate a highly effective and selective electrochemical sensor for ACV through a simple photopolymerization technique. The thin polymer layer was formed on the surface of a GCE using the template molecule ACV, along with a functional monomer (acrylamide), a basic monomer (2‐hydroxyethyl methacrylate), a cross‐linking agent (ethylene glycol dimethacrylate), and a photoinitiator (2‐hydroxy‐2‐methyl propiophenone) [82]. In a valuable study, ACV was detected electrochemically using synthesized graphitic carbon nitride (g‐C_3_N_4_). The characterization of the synthesized g‐C_3_N_4_ was performed using several techniques, including EDX, XRD, FT‐IR, FESEM, and high‐resolution transmission electron microscopy (HR‐TEM). For monitoring ACV, a composite of g‐C_3_N_4_ and pure carbon paste (g‐C_3_N_4_/CPE) was employed in voltammetric techniques [30]. The procedure for the voltammetric determination of ACV at trace levels was developed, marking the first instance of using a commercially available boron‐doped diamond electrode (BDDE) in conjunction with DPV for this application. This commercially available BDDE is noted for its quick response time, low background current, and excellent analytical performance in the determination of ACV [83]. A new nanocomposite consisting of reduced graphene oxide, palladium nanoparticles, and poly (2‐amino‐4‐chlorophenol) (rGO/Pd@PACP) was created through a chemical method to enhance the surface of a pencil graphite electrode (PGE). The effectiveness of this innovative nanomaterial‐based biosensor in analyzing interactions between drugs and DNA has been showcased [29]. The use of calcium‐doped zinc oxide (Ca‐ZnO) nanoparticles as a new electroanalytical sensor for detecting the antiviral medication ACV was investigated. Sensors modified with Ca‐ZnO nanoparticles demonstrated improved electrochemical characteristics for ACV detection when compared to an unmodified GCE [84]. A sensitive electrochemical sensor for detecting ACV was developed utilizing a glassy carbon electrode modified with a reduced graphene oxide–TiO_2_–gold (rGO–TiO_2_–Au) nanocomposite. The morphology, structure, and compositional characteristics of the rGO–TiO_2_–Au nanocomposites were verified through transmission electron microscopy, X‐ray diffraction, and X‐ray photoelectron spectroscopy [31]. An electrochemical sensor utilizing ferrous molybdate (FeMoO_4_) incorporated into graphene oxide composites was developed for the highly sensitive detection of ACV at nanomolar levels. The FeMoO_4_ nanorods were synthesized using a hydrothermal technique and subsequently combined with graphene oxide through ultrasonication. Characterization of the materials was conducted using SEM, XRD, and EDS. The sensor was constructed using a drop‐coating method to facilitate the measurement of ACV [85]. A new voltammetric nanosensor has been created for the simultaneous measurement of ACV. This sensor features a carbon paste electrode modified with nanodiamond‐coated dysprosium oxide and an ionic liquid (ND@Dy_2_O_3_‐IL/CPE). Various electrochemical techniques, including CV, chronoamperometry, EIS, and SWV, were employed to assess the performance of the modified electrodes [86]. In a particular study, techniques such as CVs, DPV, and chronoamperometry were utilized to investigate the electrochemical properties of ganciclovir on a modified electrode composed of Fe‐Cu/TiO_2_/multi‐walled CNTs/carbon paste (Fe‐Cu/TiO_2_/MWCNTs/CPE). The results indicated that the sensor we developed demonstrated stability and exhibited high sensitivity for detecting ganciclovir at physiological pH levels [87]. SERS is an effective, swift, and accurate method for detecting minute quantities of biomaterials and chemicals. In this study, filter paper substrates embedded with silver nanoparticles (AgNPs) were used as SERS biosensors to identify ACV and mitigate its negative effects. To begin with, a chemical reduction method was employed to synthesize the AgNPs [88]. A modified pencil graphite electrode, enhanced with ruthenium nanoparticles and thiobarbituric acid (RuNPs/TBA/PGE), was employed to detect ACV utilizing a highly sensitive adsorptive differential pulse voltammetric (AdDPV) method. The characterization of the modified electrode was conducted using FESEM, EIS, and CV [89]. ACV was quantitatively analyzed through electrochemical methods utilizing a carbon paste electrode modified with poly murexide and a magnetic chitosan‐curcumin composite (p.Mur‐Mag@Chi‐Cur‐A/CPE) [90]. The study explores the adsorption and transport of ACV on both unaltered and modified magnetic nanoparticles. The synthesized magnetite nanoparticles are treated with 3‐(triethoxysilyl)‐propylamine before the incorporation of ACV. Various characterization techniques, including SEM, TEM, VSM, DLS, and zeta potential analysis, are employed. Results from VSM and zeta potential measurements indicate that the presence of ACV reduces both the saturation magnetization and the zeta potential of the MNs [91]. A new voltammetric sensor has been created using a GCE that is enhanced with a thin layer of MWCNTs and an electropolymerized coating of tiron‐doped polypyrrole. This modified electrode was utilized for the detection of ACV [92]. A straightforward and effective method is introduced for the precise detection of the antiviral medication ACV using a GCE enhanced with a composite film of single‐walled CNTs and Nafion. This approach employs square wave voltammetry for the first time. The resulting sensor demonstrates strong and consistent electron transfer capabilities, producing higher peak currents at reduced potentials compared to those recorded with an unmodified GCE [93]. In a particular study, a novel sensor was developed for enhanced detection of ACV by integrating various deep eutectic solvents (DESs), zinc oxide (ZnO) nanoparticles, and MWCNTs into a carbon paste matrix. This was followed by the electropolymerization of arginine on the resulting electrode. The research also examined how key factors influenced the performance of the electrode [94]. A novel sensor was created by embedding MWCNTs and titanium dioxide nanoparticles (TiO_2_ NPs) into a polymeric matrix, which was then polymerized within a nanoporous glassy carbon electrode. The oxidation of ACV showed improved results on this modified electrode because of the combined benefits of the nanoporous structure of the glassy carbon electrode, the polymeric film, and the presence of MWCNTs and TiO_2_ NPs [95]. A highly sensitive electrochemical sensor utilizing two‐dimensional (2D) graphene nanosheets has been created for the simultaneous detection of acetaminophen and valacyclovir. This sensor was constructed by simultaneously electrochemically reducing and depositing GO onto a GCE through CV. To investigate the electrocatalytic properties of the electrochemically rGO modified GCE and to clarify the oxidation behavior of acetaminophen and valacyclovir, both CV and DPV techniques were employed [96]. A sensitive and straightforward electrochemical sensor has been developed for the quantification of ACV in human biofluids. Initially, a thin film of rGO was electrodeposited onto a PGE. Subsequently, a thin polymer layer of L‐methionine (L‐Met) was electropolymerized onto the rGO‐coated PGE, resulting in the formation of the P‐L‐Met/rGO/PGE sensor. The morphology and electrochemical performance of the modified electrode were characterized using various techniques [97]. The Supporting Information regarding the discussion of electrochemical sensors for the detection of ACV is summarized in Table 2.

**TABLE 2:** Various electrochemical sensors for the detection of ACV.

Type	Technique	Platform/NPs	Electrode	Sample/Matrix	Linear range	LOD	Sensitivity	Selectivity	Advantages	Limitations	Ref
EL	LSV	Ti_3_AlC_2_ MWCNTs‐COOH	GCE	Pharmaceutical and urine	20.0–500.0 μM	0.088 μM	High (0.088 μM)	Moderate	Good stability and reproducibility	Limited linear range	[81]
EL	MIP	AM‐ACV@MIP/GCE	GCE	Pharmaceutical	1 × 10^−11^ to 1 × 10^−10^ M	7.15 × 10^−13^ M	Very High (7.15 × 10–13 M)	High	High specificity due to molecular imprinting	Complex synthesis process	[82]
EL	EDX, XRD, FT‐IR FESEM	g‐C_3_N_4_/CPE	CPE	Serum and urine	10–80 μM	3.52 nM	High (3.52 nM)	Moderate	Effective for biological samples	Sensitivity may vary with sample matrix	[30]
EL	CVs, DPVs	—	BDDE	Pharmaceutical	0.0001–0.001 μmol L^−1^	0.0299 nmol L^−1^	Very High (0.0299 nmol L^−1^)	High	Excellent for trace detection	Potential leaching of materials	[83]
EL	CVs, DPVs	rGO/Pd@PACP	PGE	Pharmaceutical	0.1–0.5 μM	0.0513 μM	High (6.18 nM)	Moderate	Broad linear range	Stability issues under certain conditions	[29]
EL	CVs, DPVs	Ca‐ZnONPs	GCE	Pharmaceutical	8.0 × 10^−8^ M to 2.4 × 10^−5^ M	6.18 nM	High (20 nM)	Moderate	Versatile for various drug matrices	Requires careful handling	[84]
EL	CVs, LSV	rGO–TiO_2_–AuNPs	GCE	Pharmaceutical	1–100 μM	7 μL	High (0.029 μM)	High	Effective for serum analysis	Limited availability of materials	[31]
EL	EDX, XRD, FESEM	FeMoO_4_‐GO/GCE	GCE	Drug	0.1–10 μM	20 nM	Very High (0.8 nM)	High	Suitable for pharmaceutical applications	Cost of materials	[85]
EL	EDX, XRD, FESEM, SWV	ND@Dy_2_O_3_‐IL/CPE	CPE	Serum	0.097–116.6 μM	0.029 μM	Moderate	Moderate	Facilitates sample recovery	May require additional separation steps	[86]
EL	CVs, DPV, CA	Fe‐Cu/TiO_2_/MWCNTs/CPE	CPE	Real	0.1– 40 nM	10 μM	High (1.8 nM)	High	Good for urine analysis	Potential aggregation issues	[87]
EL/optical	SERS‐FPS	AgNPs	—	Biological	—	10^−12^ M	High	High	Suitable for pharmaceutical applications	Limited availability of materials	[88]
EL	CVs, EIS	RuNPs/TBA/PGE	PGE	Tablet	30.0–3000.0 nM	0.8 nM	Very high (0.007 μM)	High	Effective for human fluid analysis	Potential interference from the matrix	[89]
EL	CVs, CA	p.Mur‐Mag@Chi‐Cur‐A/CPE	CPE	Real	0.03 to 3.5 μM	7.0 nM	High (0.01 μmol dm^−3^)	Moderate	Versatile for various applications	Complexity in material synthesis	[90]
EL	TEM, VSM, DLS	MNPs‐Fe_3_O_4_	—	Pharmaceutical	—	—	Very High (1.34 nM)	High	Excellent for drug detection	Stability concerns in long‐term use	[91]
EL	LSV	MWCNT	GCE	Pharmaceutical and clinical	0.03–10.0 μM	10.0 nM	High (30 nM)	High	Suitable for human biofluids	Requires careful calibration	[92]
EL	CVs, SWV	SWNT/Naf/GCE	GCE	Urine	10–30 μM	1.8 nM	Extremely High (10^−12^ M)	Very high	Highly sensitive for biological samples	Cost and availability of silver nanoparticles	[93]
EL	DPASV	MWCNTs, ZNO	CPE	Human fluid	0.33–1.0 μM	0.007 μM	Moderate	Moderate	Facilitates sample recovery	May require additional separation steps	[94]
EL	—	MWCNTs+TiO_2_ NPs	GCE	Human fluid and tablet	0.03 to 1.0 μmol dm^−3^	0.01 μmol dm^−3^	High (0.1 μM)	High	Good for various detection applications	Potential aggregation issues	[95]
EL	CVs, DPV	rGO, GCE	GCE	Drug	—	1.34 nM	High (20 nM)	Moderate	Effective for environmental monitoring	Limited specificity for certain analytes	[96]
EL	DPV	P–L–Met/rGO/PGE	PGE	Human biofluids	0.044–2.98 μM	30 nM	High (0.01 μM)	High	Good for detecting low concentrations in complex matrices	Potential interference from other biomolecules	[97]

Table 2 presents a comprehensive summary of various electrochemical sensors developed for the detection of ACV, a widely used antiviral medication. The table categorizes the sensors on the basis of their type, technique, platform, or NPs used, electrode materials, sample matrices, linear ranges, LOD, and references. The electrochemical sensors employ a range of techniques, including linear sweep voltammetry (LSV), cyclic voltammetry (CV), differential pulse voltammetry (DPV), and others, showcasing the versatility of electrochemical methods in analytical applications. Various electrode materials, such as glassy carbon electrodes (GCE), carbon paste electrodes (CPE), and platinum group electrodes (PGE), are utilized, often enhanced with nanomaterials like MWCNTs, reduced graphene oxide (rGO), and metal NPs to improve sensitivity and selectivity. The sample matrices analyzed include pharmaceuticals, biological fluids such as serum and urine, and real human fluids, indicating the practical applicability of these sensors in clinical and pharmaceutical settings. The linear ranges of detection vary significantly, from as low as 10 nM to several hundred micromolar, reflecting the sensors' capability to detect ACV across a wide concentration spectrum. The LOD also demonstrates impressive sensitivity, with some sensors achieving LODs in the picomolar range. Overall, the advancements in electrochemical sensor technology for ACV detection highlight the potential for rapid, sensitive, and specific monitoring of this important antiviral drug, which is crucial for effective patient management and therapeutic monitoring. The references provided offer a pathway for further exploration of the methodologies and findings in this field.

## 10 Conclusion and Future Directions

In conclusion, the development of electrochemical sensors for the detection of ACV represents a significant advancement in pharmaceutical analysis, particularly given ACV's critical role as a broad‐spectrum antiviral drug against various herpes viruses. The diverse range of techniques and materials employed in these sensors, as highlighted in Table 2, underscores their potential for rapid, sensitive, and specific detection of ACV in various matrices, including pharmaceuticals and biological fluids. The impressive LOD and wide linear ranges achieved by these sensors demonstrate their applicability in clinical settings, where accurate monitoring of drug levels is essential for effective patient management. However, current limitations must be acknowledged. Issues such as selectivity in complex matrices, reusability of the sensors, and potential interference from other substances can impact the reliability of ACV detection. Addressing these challenges is crucial for the advancement of electrochemical sensors in real‐world applications. In the future, the prospects for electrochemical sensors for ACV detection are promising. Continued research and innovation in nanomaterials and electrode modifications are expected to enhance the sensitivity and selectivity of these sensors. The integration of advanced materials, such as nanocomposites and hybrid structures, could lead to more efficient sensors capable of detecting ACV at lower concentrations and in more complex matrices. Additionally, the development of portable and user‐friendly devices could facilitate point‐of‐care testing, making it easier for healthcare providers to monitor ACV levels in real time. Moreover, future work should focus on overcoming the current limitations by exploring novel electrochemical techniques, such as impedance spectroscopy and photoelectrochemical methods, which may provide new avenues for enhancing sensor performance. The combination of electrochemical methods with other analytical techniques, such as microfluidics and lab‐on‐a‐chip technologies, could also pave the way for more comprehensive and efficient detection systems. In summary, as the demand for effective monitoring of antiviral therapies continues to grow, ongoing advancements in electrochemical sensor technology will play a crucial role in improving patient outcomes and ensuring the safe and effective use of ACV and other antiviral medications. Future research should prioritize addressing current limitations, optimizing sensor designs, and expanding the range of detectable analytes to meet the evolving needs of the pharmaceutical and clinical communities.

## Author Contributions

Mohammad Darvishi, Mohammad Mahdi Heidari, and Reza Kheradmand: writing and editing original draft; Nava Moghadasian Niaki, Mahsa Tabean, and Ahmad Mobed: supervision.

## Conflicts of Interest

The authors declare no conflicts of interest.

## Data Availability

The data that support the findings of this study are available from the corresponding author upon reasonable request.

## References

[1] B. A. Taha , Y. A. Mashhadany , A. H. J. al‐Jumaily , M. S. D. B. Zan , and N. Arsad , “SARS‐CoV‐2 Morphometry Analysis and Prediction of Real Virus Levels Based on Full Recurrent Neural Network Using TEM Images,” Viruses 14, no. 11 (2022): 2386. 36366485 10.3390/v14112386 PMC9698148

[2] M. U. Kraemer , M. U. G. Kraemer , J. L.‐H. Tsui , et al., “Artificial Intelligence for Modelling Infectious Disease Epidemics,” Nature 638, no. 8051 (2025): 623–635. 39972226 10.1038/s41586-024-08564-w PMC11987553

[3] R. Gibb , D. W. Redding , S. Friant , and K. E. Jones , “Towards a ‘People and Nature’ Paradigm for Biodiversity and Infectious Disease,” Philosophical Transactions of the Royal Society, B: Biological Sciences 380, no. 1917 (2025): 20230259. 10.1098/rstb.2023.0259 PMC11712283 39780600

[4] J. Sassine , E. A. Siegrist , and R. F. Chemaly , “Herpesvirus Infections After Chimeric Antigen Receptor T‐Cell Therapy and Bispecific Antibodies: A Review,” Viruses 17, no. 1 (2025): 133. 39861922 10.3390/v17010133 PMC11768728

[5] M. M. E. Oliveira , L. B. Campos , F. Brito , et al., “Oral Microbiota and Inflammatory Bowel Diseases: Detection of Emerging Fungal Pathogens and Herpesvirus,” Biomedicine 13, no. 2 (2025): 480. 10.3390/biomedicines13020480 PMC11852465 40002893

[6] F. Budak , E. Piskin , A. Cetinkaya , and S. A. Ozkan , “Applications of Antiviral Drugs With Electrochemical Sensors,” Essential Chem 2, no. 1 (2025): 1–17.40248686

[7] S. F. Saeed and Z. A. Aldhaher , “Evaluation of Salivary Cytomegalovirus and Epstein–Barr Virus Antibody Levels in Recurrent Aphthous Stomatitis: An Analytical Cross‐Sectional Study,” Dental Hypotheses 16, no. 1 (2025): 4–6.

[8] T. G. Lagziel , M. Jurkowicz , O. Gordon , et al., “Israeli Neonatal Herpes Simplex Infection: Unique Epidemiology and Clinical Profile,” Journal of Medical Virology 96, no. 9 (2024): e29934. 39311627 10.1002/jmv.29934

[9] N. M. Niaki , F. Hatefnia , M. M. Heidari , M. Tabean , and A. Mobed , “Alpha‐Fetoprotein (AFP) Biosensors,” Clinica Chimica Acta 573 (2025): 120293. 10.1016/j.cca.2025.120293 40216053

[10] B. A. Taha , A. J. Addie , A. C. Kadhim , et al., “Photonics‐Powered Augmented Reality Skin Electronics for Proactive Healthcare: Multifaceted Opportunities,” Microchimica Acta 191, no. 5 (2024): 250. 38587660 10.1007/s00604-024-06314-3

[11] B. A. Taha , A. J. Addie , A. C. Kadhim , et al., “Plasmonic‐Enabled Nanostructures for Designing the Next Generation of Silicon Photodetectors: Trends, Engineering and Opportunities,” Surfaces and Interfaces 48 (2024): 104334.

[12] B. Ahmed Taha , A. C. Kadhim , A. J. Addie , et al., “Advancing Cancer Diagnostics Through Multifaceted Optical Biosensors Supported by Nanomaterials and Artificial Intelligence: A Panoramic Outlook,” Microchemical Journal 205 (2024): 111307.

[13] E. Piperi , E. Papadopoulou , M. Georgaki , et al., “Management of Oral Herpes Simplex Virus Infections: The Problem of Resistance. A Narrative Review,” Oral Diseases 30, no. 3 (2024): 877–894. 37279074 10.1111/odi.14635

[14] L. Wilms , K. Weßollek , T. B. Peeters , and A. S. Yazdi , “Infections With Herpes Simplex and Varicella Zoster Virus,” Journal of the German Society of Dermatology 20, no. 10 (2022): 1327–1351. 10.1111/ddg.14917 36184818

[15] A. L. Verzosa , L. A. McGeever , S. J. Bhark , T. Delgado , N. Salazar , and E. L. Sanchez , “Herpes Simplex Virus 1 Infection of Neuronal and Non‐Neuronal Cells Elicits Specific Innate Immune Responses and Immune Evasion Mechanisms,” Frontiers in Immunology 12 (2021): 644664. 34135889 10.3389/fimmu.2021.644664 PMC8201405

[16] Y. Iwaisako and M. Fujimuro , “The Terminase Complex of Each Human Herpesvirus,” Biological and Pharmaceutical Bulletin 47, no. 5 (2024): 912–916. 38692868 10.1248/bpb.b23-00717

[17] T. J. Hill , “Herpes Simplex Virus Latency,” in The Herpesviruses: Volume 3, ed. B. Roizman (Springer US, 1985), 175–240.

[18] K. Kondo , T. Kondo , T. Okuno , M. Takahashi , and K. Yamanishi , “Latent Human Herpesvirus 6 Infection of Human Monocytes/Macrophages,” Journal of General Virology 72, no. 6 (1991): 1401–1408. 1646280 10.1099/0022-1317-72-6-1401

[19] M. Luppi , P. Barozzi , C. Morris , et al., “Human Herpesvirus 6 Latently Infects Early Bone Marrow Progenitors In Vivo,” Journal of Virology 73, no. 1 (1999): 754–759. 9847383 10.1128/jvi.73.1.754-759.1999 PMC103884

[20] J. I. Cohen , “Epstein–Barr Virus Infection,” New England Journal of Medicine 343, no. 7 (2000): 481–492. 10944566 10.1056/NEJM200008173430707

[21] K. Kardani , J. Sanchez Gil , and S. D. Rabkin , “Oncolytic Herpes Simplex Viruses for the Treatment of Glioma and Targeting Glioblastoma Stem‐Like Cells,” Frontiers in Cellular and Infection Microbiology 13 (2023): 1206111. 37325516 10.3389/fcimb.2023.1206111 PMC10264819

[22] A. Ärlemalm , A. Helldén , L. Karlsson , and B. Carlsson , “Rapid Determination of Acyclovir, Its Main Metabolite 9‐Carboxymethoxymethylguanine, Ganciclovir, and Penciclovir in Human Serum Using LC–MS/MS,” Biomedical Chromatography 36, no. 4 (2022): e5315. 34981553 10.1002/bmc.5315 PMC9285573

[23] A. Rousseau , S. B. Pharm , J. Gueudry , et al., “Acyclovir‐Resistant Herpes Simplex Virus 1 Keratitis: A Concerning and Emerging Clinical Challenge,” American Journal of Ophthalmology 238 (2022): 110–119. 35033543 10.1016/j.ajo.2022.01.010

[24] S. M. Awad , S. M. Ali , Y. E. Mansour , and S. S. Fatahala , “Synthesis and Evaluation of Some Uracil Nucleosides as Promising Anti‐Herpes Simplex Virus 1 Agents,” Molecules 26, no. 10 (2021): 2988. 34069874 10.3390/molecules26102988 PMC8157375

[25] V. Andronova , G. A. Galegov , O. A. Vozdvizhenskaya , G. L. Levit , V. P. Krasnov , and V. N. Charushin , “Combined Effect of Basic Antiherpetic Drugs With a New Inhibitor of the Terminase Complex of Herpes Simplex Virus Type 1 in Vero Cell Cultures,” Doklady Biological Sciences (2024): 55–58. 38955885 10.1134/S0012496624701035

[26] F. Heidary , S. Madani , R. Gharebaghi , and F. Asadi‐Amoli , “Acyclovir as a Potential Add‐on Therapy in COVID‐19 Treatment Regimens,” Pharmaceutical Sciences 27 (2021): S68–S77.

[27] M. Chakravarty and A. Vora , “Nanotechnology‐Based Antiviral Therapeutics,” Drug Delivery and Translational Research 11, no. 3 (2021): 748–787. 32748035 10.1007/s13346-020-00818-0 PMC7398286

[28] Y. Shi , D. He , X. Zhang , M. Yuan , and X. Liu , “Research Progress in Nanopharmaceuticals With Different Delivery Routes in the Antivirus Field,” Current Pharmaceutical Design 29, no. 25 (2023): 1975–1991. 37644796 10.2174/1381612829666230830105817

[29] S. Yanik , D. Emre , M. Alp , et al., “A Novel Electrochemical Biosensor Based on Palladium Nanoparticles Decorated on Reduced Graphene Oxide‐Polyaminophenol Matrix for the Detection and Discrimination of Mitomycin C‐DNA and Acyclovir‐DNA Interaction,” Journal of Pharmaceutical and Biomedical Analysis 234 (2023): 115524. 37320972 10.1016/j.jpba.2023.115524

[30] M. Sreenivasulu , S. J. Malode , S. A. Alqarni , and N. P. Shetti , “Graphitic Carbon Nitride (g–C3N4)‐based Electrochemical Sensors for the Determination of Antiviral Drug Acyclovir,” Materials Chemistry and Physics 312 (2024): 128650.

[31] X.‐Y. Lu , J. Li , F. Y. Kong , et al., “Improved Performance for the Electrochemical Sensing of Acyclovir by Using the rGO–TiO_2_–Au Nanocomposite‐Modified Electrode,” Frontiers in Chemistry 10 (2022): 892919. 35646815 10.3389/fchem.2022.892919 PMC9130495

[32] Y.‐P. Wei , L. Y. Yao , Y. Y. Wu , et al., “Critical Review of Synthesis, Toxicology and Detection of Acyclovir,” Molecules 26, no. 21 (2021): 6566. 34770975 10.3390/molecules26216566 PMC8587948

[33] M. Sultan , “Spectrophotometric Determination of Acyclovir in Some Pharmaceutical Formulations,” Il Farmaco 57, no. 11 (2002): 865–870. 12484534 10.1016/s0014-827x(02)01299-5

[34] U. Ajima and J. O. Onah , “Spectrophotometric Determination of Acyclovir After Its Reaction With Ninhydrin and Ascorbic Acid,” Journal of Applied Pharmaceutical Science 5 (2015): 065–069.

[35] T. A. Kumar , B. M. Gurupadayya , M. R. Reddy , and M. P. Raju , “Selective and Validated Spectrophotometric Method for Determination of Acyclovir and Valacyclovir Using N‐Bromosuccinimide,” Journal of Pharmacy Research 4, no. 1 (2011): 24–27.

[36] G. Bahrami , S. Mirzaeei , and A. Kiani , “Determination of Acyclovir in Human Serum by High‐Performance Liquid Chromatography Using Liquid–Liquid Extraction and Its Application in Pharmacokinetic Studies,” Journal of Chromatography B 816, no. 1–2 (2005): 327–331. 10.1016/j.jchromb.2004.11.038 15664366

[37] A. Šmidovnik , A. G. Wondra , and M. Prošek , “Determination of Acyclovir in Plasma by High Performance Liquid Chromatography With UV Detection. Method Development and Method Validation,” Journal of High Resolution Chromatography 20, no. 9 (1997): 503–506.

[38] S. D. Brown , C. A. White , C. K. Chu , and M. G. Bartlett , “Determination of Acyclovir in Maternal Plasma, Amniotic Fluid, Fetal and Placental Tissues by High‐Performance Liquid Chromatography,” Journal of Chromatography B 772, no. 2 (2002): 327–334. 10.1016/s1570-0232(02)00120-4 12007778

[39] L. Zeng , C. E. Nath , P. J. Shaw , J. W. Earl , and A. J. McLachlan , “HPLC‐Fluorescence Assay for Acyclovir in Children,” Biomedical Chromatography 22, no. 8 (2008): 879–887. 18348336 10.1002/bmc.1006

[40] A. Maes , B. Garré , N. Desmet , et al., “Determination of Acyclovir in Horse Plasma and Body Fluids by High‐Performance Liquid Chromatography Combined With Fluorescence Detection and Heated Electrospray Ionization Tandem Mass Spectrometry,” Biomedical Chromatography 23, no. 2 (2009): 132–140. 18823074 10.1002/bmc.1093

[41] P. D. Tzanavaras and D. G. Themelis , “High‐Throughput HPLC Assay of Acyclovir and Its Major Impurity Guanine Using a Monolithic Column and a Flow Gradient Approach,” Journal of Pharmaceutical and Biomedical Analysis 43, no. 4 (2007): 1526–1530. 17142000 10.1016/j.jpba.2006.11.002

[42] D. Zendelovska , S. Simeska , E. Atanasovska , et al., “Determination of Acyclovir in Human Plasma Samples by HPLC Method With UV Detection: Application to Single‐Dose Pharmacokinetic Study,” Open Access Macedonian Journal of Medical Sciences 3, no. 1 (2015): 32–36. 27275193 10.3889/oamjms.2015.011 PMC4877785

[43] R. Urinovska , I. Kacirova , and J. Sagan , “Determination of Acyclovir and Its Metabolite 9‐Carboxymethoxymethylguanide in Human Serum by Ultra‐High‐Performance Liquid Chromatography‐Tandem Mass Spectrometry,” Journal of Separation Science 44, no. 16 (2021): 3080–3088. 34165890 10.1002/jssc.202100241

[44] D. Schimek , R. Raml , K. A. Francesconi , M. Bodenlenz , and F. Sinner , “Quantification of Acyclovir in Dermal Interstitial Fluid and Human Serum by Ultra‐High‐Performance Liquid–High‐Resolution Tandem Mass Spectrometry for Topical Bioequivalence Evaluation,” Biomedical Chromatography 32, no. 6 (2018): e4194. 29349796 10.1002/bmc.4194

[45] V. Mulabagal , M. Annaji , S. Kurapati , et al., “Stability‐Indicating HPLC Method for Acyclovir and Lidocaine in Topical Formulations,” Biomedical Chromatography 34, no. 3 (2020): e4751. 31756271 10.1002/bmc.4751

[46] D. Bitas and V. Samanidou , “Molecularly Imprinted Polymers as Extracting Media for the Chromatographic Determination of Antibiotics in Milk,” Molecules 23, no. 2 (2018): 316. 29393877 10.3390/molecules23020316 PMC6017535

[47] Y. Wu , P. Deng , Y. Tian , et al., “Rapid Recognition and Determination of Tryptophan by Carbon Nanotubes and Molecularly Imprinted Polymer‐Modified Glassy Carbon Electrode,” Bioelectrochemistry 131 (2020): 107393. 31698180 10.1016/j.bioelechem.2019.107393

[48] S. Wu , L. Tan , G. Wang , G. Peng , C. Kang , and Y. Tang , “Binding Characteristics of Homogeneous Molecularly Imprinted Polymers for Acyclovir Using an (Acceptor–Donor–Donor)—(Donor–Acceptor–Acceptor) Hydrogen‐Bond Strategy, and Analytical Applications for Serum Samples,” Journal of Chromatography A 1285 (2013): 124–131. 23489483 10.1016/j.chroma.2013.02.039

[49] H. Yan , M. Wang , Y. Han , F. Qiao , and K. H. Row , “Hybrid Molecularly Imprinted Polymers Synthesized With 3‐Aminopropyltriethoxysilane‐Methacrylic Acid Monomer for Miniaturized Solid‐Phase Extraction: A New and Economical Sample Preparation Strategy for Determination of Acyclovir in Urine,” Journal of Chromatography A 1346 (2014): 16–24. 24811152 10.1016/j.chroma.2014.04.045

[50] M. Tang , B. T. Zhang , Y. Teng , M. Liu , and Y. Zhang , “Fast Determination of Peroxymonosulfate by Flow Injection Chemiluminescence Using the Tb (III) Ligand in Micelle Medium,” Luminescence 35, no. 2 (2020): 274–283. 31736184 10.1002/bio.3724

[51] M. Asghar , F. Ameen , S. Al‐Nadhari , et al., “A Flow Injection Chemiluminescence Method for the Determination of Retinol in Pharmaceutical Formulations by Using Luminol‐Diperiodatoargentate (III) Reaction,” Journal of Nutritional Science and Vitaminology 66, no. 1 (2020): 10–18. 32115448 10.3177/jnsv.66.10

[52] X. Long and F. Chen , “Flow Injection–Chemiluminescence Determination of Acyclovir,” Luminescence 27, no. 6 (2012): 478–481. 22223601 10.1002/bio.1378

[53] K. Kłysik , A. Pietraszek , A. Karewicz , and M. Nowakowska , “Acyclovir in the Treatment of Herpes Viruses–A Review,” Current Medicinal Chemistry 27, no. 24 (2020): 4118–4137. 29521211 10.2174/0929867325666180309105519

[54] A. Birkmann , S. Bonsmann , D. Kropeit , et al., “Discovery, Chemistry, and Preclinical Development of Pritelivir, a Novel Treatment Option for Acyclovir‐Resistant Herpes Simplex Virus Infections,” Journal of Medicinal Chemistry 65, no. 20 (2022): 13614–13628. 36202389 10.1021/acs.jmedchem.2c00668 PMC9620171

[55] L. Brower , A. Schondelmeyer , P. Wilson , and S. S. Shah , “Testing and Empiric Treatment for Neonatal Herpes Simplex Virus: Challenges and Opportunities for Improving the Value of Care,” Hospital Pediatrics 6, no. 2 (2016): 108–111. 26740558 10.1542/hpeds.2015-0166

[56] E. Khezerloo , F. Hekmat , A. Iraji zad , and S. Shahrokhian , “A Novel Electrochemical Probe Crafted From Copper Oxide Nanoball‐Multiwalled Carbon Nanotube for Valacyclovir Detection in Environmental and Medicinal Matrices,” Electrochimica Acta 537 (2025): 146872.

[57] Y. Hayat , M. Tariq , A. Hussain , and A. Tariq , “A Review of Biosensors and Artificial Intelligence in Healthcare and Their Clinical Significance,” International Research Journal of Economics and Management Studies 3, no. 1 (2024): 230–247.

[58] C. M. Almeida , B. Merillas , and A. D. R. Pontinha , “Trends on Aerogel‐Based Biosensors for Medical Applications: An Overview,” International Journal of Molecular Sciences 25, no. 2 (2024): 1309. 38279307 10.3390/ijms25021309 PMC10816975

[59] T. Wasilewski , W. Kamysz , and J. Gębicki , “AI‐Assisted Detection of Biomarkers by Sensors and Biosensors for Early Diagnosis and Monitoring,” Biosensors 14, no. 7 (2024): 356. 39056632 10.3390/bios14070356 PMC11274923

[60] O. B. Daramola , R. K. Omole , and B. A. Akinsanola , “Emerging Applications of Biorecognition Elements‐Based Optical Biosensors for Food Safety Monitoring,” Discover Sensors 1, no. 1 (2025): 3.

[61] M. Tewari , P. Rana , and V. Pande , “Nanomaterial‐Based Biosensors for the Detection of COVID‐19,” Indian Journal of Microbiology 65, no. 1 (2024): 120–136. 40371045 10.1007/s12088-024-01336-0 PMC12069788

[62] A. Ait Lahcen and A. Amine , “Biorecognition elements,” in Wearable Physical, Chemical and Biological Sensors (Elsevier, 2022), 41–70.

[63] T. D. Pollard , J. J. Ong , A. Goyanes , et al., “Electrochemical Biosensors: A Nexus for Precision Medicine,” Drug Discovery Today 26, no. 1 (2021): 69–79. 33137482 10.1016/j.drudis.2020.10.021

[64] A. Haleem , M. Javaid , R. P. Singh , R. Suman , and S. Rab , “Biosensors Applications in Medical Field: A Brief Review,” Sensors International 2 (2021): 100100.

[65] A. Singh , A. Sharma , A. Ahmed , et al., “Recent Advances in Electrochemical Biosensors: Applications, Challenges, and Future Scope,” Biosensors 11, no. 9 (2021): 336. 34562926 10.3390/bios11090336 PMC8472208

[66] L. Zhang , W. Guo , C. Lv , et al., “Electrochemical Biosensors Represent Promising Detection Tools in Medical Field,” Advanced Sensor and Energy Materials 2, no. 4 (2023): 100081.

[67] J. Brindha , M. S. Dangate , and M. Balamurali , “Environment Remediation Tools: Chemosensors and Biosensors,” in Environmental Pollution and Remediation (Springer Singapore, 2021), 267–293.

[68] M. Xu , A Bifunctional Nanocomposites Based Electrochemical Biosensor for In‐Field Detection of Pathogenic Bacteria in Food (University of Arkansas, 2016).

[69] S. Marchenko , O. Saiapina , Y. Nesterenko , et al., “A Novel Conductometric Biosensor Based on Hybrid Organic/Inorganic Recognition Element for Determination of L‐Arginine,” Bioelectrochemistry 165 (2025): 108977. 40245598 10.1016/j.bioelechem.2025.108977

[70] N. Sitkov , A. Ryabko , V. Moshnikov , A. Aleshin , D. Kaplun , and T. Zimina , “Hybrid Impedimetric Biosensors for Express Protein Markers Detection,” Micromachines 15, no. 2 (2024): 181. 38398911 10.3390/mi15020181 PMC10890403

[71] Y.‐C. Ouyang , B. J. Yeom , Y. Zhao , and W. Ma , “Progress and Prospects of Chiral Nanomaterials for Biosensing Platforms,” Rare Metals 43, no. 6 (2024): 2469–2497.

[72] A. Karnwal , R. S. Kumar Sachan , I. Devgon , et al., “Gold Nanoparticles in Nanobiotechnology: From Synthesis to Biosensing Applications,” ACS Omega 9, no. 28 (2024): 29966–29982. 39035946 10.1021/acsomega.3c10352 PMC11256298

[73] R. Abdel‐Karim , “Nanotechnology‐Enabled Biosensors: A Review of Fundamentals, Materials, Applications, Challenges, and Future Scope,” Biomedical Materials & Devices 2, no. 2 (2024): 759–777.

[74] H. Wang , P. Liu , J. Peng , H. Yu , and L. Wang , “Poly (3, 4‐Ethylenedioxythiophene): Poly (Styrene Sulfonate) Modified Metal‐Organic Frameworks Boosting Carbon Dots Electrochemiluminescence Emission for Sensitive miRNA Detection,” Biosensors and Bioelectronics 249 (2024): 116015. 38211464 10.1016/j.bios.2024.116015

[75] A. Bisht , A. Mishra , H. Bisht , and R. M. Tripathi , “Nanomaterial Based Biosensors for Detection of Viruses Including SARS‐CoV‐2: A Review,” Journal of Analysis and Testing 5, no. 4 (2021): 327–340. 34777896 10.1007/s41664-021-00200-0 PMC8572656

[76] G. Manoharan , P. Bösel , J. Thien , et al., “Click‐Functionalization of Silanized Carbon Nanotubes: From Inorganic Heterostructures to Biosensing Nanohybrids,” Molecules 28, no. 5 (2023): 2161. 36903408 10.3390/molecules28052161 PMC10004328

[77] J. Mondal , J. M. An , S. S. Surwase , et al., “Carbon Nanotube and Its Derived Nanomaterials Based High Performance Biosensing Platform,” Biosensors 12, no. 9 (2022): 731. 36140116 10.3390/bios12090731 PMC9496036

[78] S. Malik , J. Singh , R. Goyat , et al., “Nanomaterials‐Based Biosensor and Their Applications: A Review,” Heliyon 9, no. 9 (2023): e19929. 37809900 10.1016/j.heliyon.2023.e19929 PMC10559358

[79] I. U. Hassan , G. A. Naikoo , F. Arshad , et al., “Applications of Trimetallic Nanomaterials as Non‐Enzymatic Glucose Sensors,” Drug Development and Industrial Pharmacy 49, no. 6 (2023): 393–404. 37272678 10.1080/03639045.2023.2221737

[80] H. Jiang , J. Yang , W. Wang , H. Fang , and J. Chen , “Resorcinol–Formaldehyde Resin and Sodium Dodecyl Sulfate Co‐Assisted Mesoporous TiO_2_ Supported Ni_3_Sn_2_ Intermetallic Compound Catalysts for In‐Situ Hydrodeoxygenation of Methyl Palmitate With Methanol as the Hydrogen Donor in Water,” Catalysis Surveys from Asia 29, no. 3 (2025): 1–248.

[81] N. Tang , A. Chen , Y. Wei , et al., “Highly Sensitive Determination of Acyclovir Using Ti3C2‐CS‐MWCNTs‐COOH/GCE Composite as a Sensing Platform,” Colloids and Surfaces A: Physicochemical and Engineering Aspects 695 (2024): 134248.

[82] A. Al Faysal , A. Cetinkaya , S. I. Kaya , T. Erdoğan , S. A. Ozkan , and A. Gölcü , “Development and Fabrication of a Molecularly Imprinted Polymer‐Based Electroanalytical Sensor for the Determination of Acyclovir,” ACS Omega 9, no. 8 (2024): 9564–9576. 38434833 10.1021/acsomega.3c09399 PMC10905707

[83] D. Gorylewski , K. Tyszczuk‐Rotko , M. Wójciak , and I. Sowa , “Fast, Simple, and Sensitive Voltammetric Measurements of Acyclovir in Real Samples via Boron‐Doped Diamond Electrode,” Materials 17, no. 18 (2024): 4480. 39336222 10.3390/ma17184480 PMC11433364

[84] D. Ilager , N. P. Shetti , R. S. Malladi , N. S. Shetty , K. R. Reddy , and T. M. Aminabhavi , “Synthesis of ca‐Doped ZnO Nanoparticles and Its Application as Highly Efficient Electrochemical Sensor for the Determination of Anti‐Viral Drug, Acyclovir,” Journal of Molecular Liquids 322 (2021): 114552.

[85] Y. Wei , L. Yao , Y. Wu , et al., “Ultrasensitive Electrochemical Detection for Nanomolarity Acyclovir at Ferrous Molybdate Nanorods and Graphene Oxide Composited Glassy Carbon Electrode,” Colloids and Surfaces A: Physicochemical and Engineering Aspects 641 (2022): 128601.

[86] M. Ghadirinataj , S. K. Hassaninejad‐Darzi , and H. Emadi , “An Electrochemical Nanosensor for Simultaneous Quantification of Acetaminophen and Acyclovir by ND@Dy2O3‐IL/CPE,” Electrochimica Acta 450 (2023): 142274.

[87] T. Alinejad , C. S. Chen , M. Shamsipur , M. Bagher Gholivand , and G. Paimard , “Electrochemical Evaluation and Determination of Antiretroviral Drug Ganciclovir Based on Fe‐Cu/TiO_2_/Multi‐Walled Carbon Nanotubes Sensor,” Measurement 214 (2023): 112846.

[88] V. Eskandari , H. Sahbafar , E. Karooby , et al., “Surface‐Enhanced Raman Scattering (SERS) Filter Paper Substrates Decorated With Silver Nanoparticles for the Detection of Molecular Vibrations of Acyclovir Drug,” Spectrochimica Acta Part A: Molecular and Biomolecular Spectroscopy 298 (2023): 122762. 37130482 10.1016/j.saa.2023.122762

[89] M. Lalei and K. Zarei , “Fabrication of RuNPs/TBA/PGE and Its Application for the Electrochemical Determination of Trace Amounts of Acyclovir,” Microchemical Journal 190 (2023): 108667.

[90] M. Abd‐Elsabour , M. M. Abou‐Krisha , A. G. Alhamzani , and E. A. Abdelrahman , “A Novel Electrochemical Sensor Employing Electropolymerized Murexide on Modified Carbon Paste Electrode With Magnetic Chitosan‐Curcumin Analogue for Highly Sensitive Analysis of Acyclovir,” Journal of the Electrochemical Society 172, no. 4 (2025): 47506.

[91] X. Xie , L. Zhang , W. Zhang , et al., “Fabrication of Temperature and pH Sensitive Decorated Magnetic Nanoparticles as Effective Biosensors for Targeted Delivery of Acyclovir Anti‐Cancer Drug,” Journal of Molecular Liquids 309 (2020): 113024.

[92] S. Shahrokhian , M. Azimzadeh , and M. K. Amini , “Modification of Glassy Carbon Electrode With a Bilayer of Multiwalled Carbon Nanotube/Tiron‐Doped Polypyrrole: Application to Sensitive Voltammetric Determination of Acyclovir,” Materials Science and Engineering: C 53 (2015): 134–141. 26042700 10.1016/j.msec.2015.04.030

[93] P. Tarlekar , A. Khan , and S. Chatterjee , “Nanoscale Determination of Antiviral Drug Acyclovir Engaging Bifunctionality of Single Walled Carbon Nanotubes – Nafion Film,” Journal of Pharmaceutical and Biomedical Analysis 151 (2018): 1–9. 29291454 10.1016/j.jpba.2017.12.006

[94] M. Hamtak , L. Fotouhi , M. Hosseini , and P. Seyed Dorraji , “Improved Performance for Acyclovir Sensing in the Presence of Deep Eutectic Solvent and Nanostructures and Polymer,” IEEE Sensors Journal 20, no. 2 (2020): 623–630.

[95] M. Hamtak , L. Fotouhi , M. Hosseini , and P. S. Dorraji , “Sensitive Determination of Acyclovir in Biological and Pharmaceutical Samples Based on Polymeric Film Decorated With Nanomaterials on Nanoporous Glassy Carbon Electrode,” Journal of the Electrochemical Society 165, no. 13 (2018): B632–B637.

[96] B.‐R. Adhikari , M. Govindhan , H. Schraft , and A. Chen , “Simultaneous and Sensitive Detection of Acetaminophen and Valacyclovir Based on Two Dimensional Graphene Nanosheets,” Journal of Electroanalytical Chemistry 780 (2016): 241–248.

[97] H. Razmi , Z. Khorablou , Z. Ayazi , and F. Shahdost‐Fard , “Selective Detection of Acyclovir on Poly(L–Methionine) Membrane Coated Reduced Graphene Oxide‐Based Graphite Electrode Optimized by Central Composite Design,” IEEE Sensors Journal 21, no. 3 (2021): 2476–2484.

